# c-Jun N-Terminal Kinase (JNK) Inhibitor IQ-1S as a Suppressor of Tumor Spheroid Growth

**DOI:** 10.3390/molecules30214278

**Published:** 2025-11-03

**Authors:** Elena Afrimzon, Mordechai Deutsch, Maria Sobolev, Naomi Zurgil, Andrei I. Khlebnikov, Mikhail A. Buldakov, Igor A. Schepetkin

**Affiliations:** 1 The Biophysical Interdisciplinary Jerome Schottenstein Center for the Research and Technology of the Cellome, Physics Department, Bar Ilan University, Ramat Gan 5290002, Israel; afrimzone@gmail.com (E.A.); motti.jsc@gmail.com (M.D.); eweryday@yahoo.com (M.S.); zurgiln@gmail.com (N.Z.); 2 Institute of Nanotechnology and Advanced Materials, The Mina and Everard Goodman Faculty of Life Sciences, Bar-Ilan University, Ramat Gan 5290002, Israel; 3 Kizhner Research Center, Tomsk Polytechnic University, Tomsk 634050, Russia; 4 Cancer Research Institute, Tomsk National Research Medical Center, Russian Academy of Sciences, Tomsk 634050, Russia; buldakov@oncology.tomsk.ru; 5 Department of Microbiology and Cell Biology, Bozeman, MT 59717, USA

**Keywords:** c-Jun N-terminal kinase, kinase inhibitor, aryl oxime, MCF7 breast cancer cells, tumor spheroids

## Abstract

c-Jun N-terminal kinase (JNK) activation has been shown to play a crucial role in the development of various types of cancer. IQ-1S is a JNK inhibitor based on the 11*H*-indeno[1,2-*b*]quinoxalin-11-one scaffold. The aim of this study was to investigate the antiproliferative effect of IQ-1S on MCF7 breast cancer cells in both two-dimensional (2D) monolayer and 3D multicellular spheroid test-systems. Non-adherent, non-tethered 3D objects were generated from single MCF7 breast cancer cells in a hydrogel array. IQ-1S was added directly to the cells seeded in the hydrogel array. MCF7 spheroids were grown for 7 days. Spheroid size, growth rate, and morphology were assessed at single-object resolution. The study revealed significant differences in the size, morphology and some vital characteristics of breast cancer 3D objects when treated with the JNK inhibitor compared to vehicle (dimethyl sulfoxide)-treated controls. Spheroids treated with IQ-1S (20 μM) after 7 days are significantly smaller than the control objects. This difference was not attributable to variations in the initial number of cells seeding for the spheroid formation. Morphological examinations showed that 3D multicellular objects grown from IQ-1S-treated cells lose their regular, round morphology, in contrast to control spheroids. Furthermore, cell proliferation measured using a label-free impedance monitoring platform was reduced in monolayer (2D) culture of MCF7 cells in the presence of 10 and 20 μM IQ-1S. MCF7 cells in 2D culture treated with IQ-1S (20 μM) for 72 and 153 h showed a significant increase in apoptosis as assessed by flow cytometry with annexin V/propidium iodide staining. An in silico evaluation showed that compound IQ-1S has generally satisfactory ADME (absorption, distribution, metabolism, and excretion) properties and high bioavailability. We conclude that IQ-1S effectively inhibits the growth of 3D spheroids and MCF7 cells in 2D culture and has a high potential for use in preclinical tumor growth models.

## 1. Introduction

c-Jun N-terminal kinase (JNK) is an important regulator of mitosis, apoptosis, expression of matrix metalloproteinases, and cancer cell migration [1,2]. Activation of the JNK signaling pathway has a pivotal role in the maintenance of ovarian cancer stem cells [3], and recent studies have revealed that the JNK pathway activation is associated with a shorter progression-free survival of patients with ovarian cancer [4,5,6]. Constitutive JNK activation has been reported in gliomas and correlates with histological grade and epidermal growth factor receptor (EGFR) expression in diffuse gliomas [7,8]. The JNK activation has been shown to have a crucial role in the tumorigenesis of gliomas, possibly through the EGFR-mediated signaling pathway and maintenance of stemness [9,10,11]. Pharmacological inhibition of the JNK pathway suppressed soft-agar colony formation of sphere-forming glioma cells [11]. The increased activation of the JNK pathway enhanced multi-drug resistance in human colon cancer cells [12,13]. Targeting JNK has been reported to be beneficial in a variety of experimental tumor/metastasis models [14,15]. For example, the covalent JNK inhibitor, JNK-IN-8, suppresses triple-negative breast cancer (TNBC) growth both in vitro and in vivo [16]. Likewise, JNK inhibitor SP600125 significantly inhibited the growth of MCF7 breast cancer cells [17], steam-like glioblastoma cells [14] and spheroids from thyroid cancers [18]. Thus, JNK may prove to be a promising target for cancer treatment.

Nowadays, three-dimensional (3D) multicellular models in vitro are widely used in order to better recapitulate the morphologic and functional properties of the tumor tissue. The most important advantages of 3D multicellular object culturing is its closer likeness to the native cellular arrangement, which allows overcoming structural, physiological and molecular alterations in the cells, due to non-natural cultural conditions in vitro [19,20,21]. Three-dimensional tissue-like multicellular objects maintain cell-to-cell contacts, secrete extracellular matrix components, develop active molecule transfer gradients and respond well to microenvironmental signals, including drug treatment [22,23,24]. Two common methods of 3D object formation in vitro are known in terms of the initiating process: objects are formed by cell self-aggregation followed by cell proliferation; or multicellular objects are clones grown each from a single cell [25]. Multicellular cancer models, providing intermediate complexity between standard monolayer culture and tumors in vivo, can improve and accelerate the development and testing of new drugs and therapeutic options [26,27].

Selective inhibitors of various kinases, including cyclin-dependent kinases 4 and 6 (CDK4/6), epidermal growth factor receptor (EGFR) tyrosine kinase, and aurora kinase A have shown promise in various malignancies by inhibiting cell cycle progression and suppressing the growth of 3D tumor spheroids [28,29,30]. Aryl oximes have been reported to exhibit a high affinity for various kinases and demonstrate significant anticancer potential [31,32,33]. Previously, the sodium salt of 11*H*-indeno[1,2-*b*]quinoxalin-11-one oxime (IQ-1S) and its analogs demonstrated a high-affinity JNK inhibition in nanomolar range (K_d_ from 90 to 390 nM toward JNK1-3 isoforms) as well as the inhibition of transcription factors nuclear factor κB (NF-κB) and activator protein 1 (AP-1) activation in human cells of monocytic leukemia [34,35,36].

In the present work, effects of IQ-1S on cancer tumor growth are now studied in vitro regarding the formation and growth of the multicellular 3D breast cancer models. Non-adherent, non-tethered 3D objects were generated from single MCF7 breast cancer cells within a hydrogel array. Size, growth rate, and morphology of the spheroids were measured at single-object resolution during 7 days in the presence of IQ-1S. The study revealed significant differences in 3D breast cancer object size, morphology and some vital features under the IQ-1S treatment in comparison to control objects treated by vehicle (dimethyl sulfoxide, DMSO). The 7-day spheroids treated by IQ-1S were significantly smaller than the control objects. This difference was not attributable to the initial number of cells that seeded for the spheroid formation. Morphological examination revealed that 3D multicellular spheroids derived from IQ-1S-treated cells lost their regular, round morphology, unlike control spheroids. Experiments using MCF7 cells cultured under 2D conditions also revealed a significant reduction in the proliferation and induction of apoptosis following treatment with IQ-1S. Furthermore, computational analyses indicated that IQ-1S complies with key bioavailability criteria. Taken together, these findings suggest that IQ-1S exhibits favorable drug-like properties and medicinal chemistry compatibility and effectively inhibits the growth of MCF7 breast cancer cells in both 2D and 3D culture systems.

## 2. Results and Discussion

We used the JNK inhibitor IQ-1S [35] to study the effect of JNK inhibition on the growth of MCF7 breast cancer cells in a 3D structure format. MCF7 cells were seeded in hydrogel Microchamber Array (hMCA), allowing the 3D objects to grow for a week. We found that IQ-1S-treated multicellular objects grew more slowly than vehicle-treated control 3D spheroids. Morphological examinations showed that 3D objects grown from IQ-1S-treated cells lose their rounded shape, unlike control spheroids. However, the edges of the objects remained smooth and even. The cross-sectional area (CSA) of 7-day-old 3D objects was half that of the controls, despite the fact that the CSA of early cell conglomerates formed overnight after cell seeding was the same in both cases. In addition, initiating 3D spheroid growth with the same number of cells eliminated variability arising from differences in cell number per individual spheroid (Table 1).

**Figure 1:**
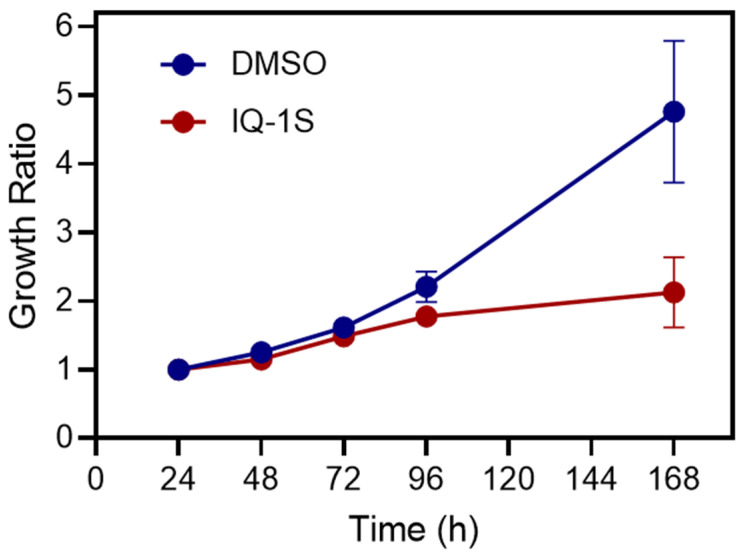
Growth kinetics of MCF7 spheroids treated with IQ-1S. The kinetics of 3D growth were estimated as the ratio of the CSA of multicellular objects treated with IQ-1S (20 μM, red circles) and untreated (0.1% DMSO, blue circles) at each time point to the area 24 h after cell seeding and presented as the mean ± standard error (S.E.).

Moreover, when the kinetics of the 3D objects were assessed as a growth ratio, i.e., as the ratio of the CSA of IQ-1S-treated and untreated multicellular objects at each time point to the area 24 h after cell seeding, it became evident that the difference in the CSA ratio increased as the growth period continued (Figure 1) and reached a maximum at the end of the experiment. IQ-1S at a low concentration (2 µM) did not affect the growth of the 3D objects: the final CSA was similar to that of control spheroids (0.008 ± 0.001 mm^2^ and 0.0076 ± 0.0030 mm^2^, *p*-value = 0.6).

In order to assess the vital features of the 3D objects, 7-day multicellular objects were double-stained with propidium iodide (PI) and fluorescein-diacetate (FDA) (final concentrations of the dyes were 3.7 μM and 1.2 μM, respectively) for the simultaneous measurement of dead and live cells per single 3D object. All live fluorescent staining procedures were performed in situ while multicellular spheroids were still within the picowell array where they grew. The level of cell death estimated as a PI-positive area was found to be higher in the 3D objects grown under the IQ-1S treatment (Table 2) in comparison to the control objects, although the difference was not significant. It should be noted that spheroids have a layered cellular structure with an outer proliferating, and inner quiescent zone, potentially with a necrotic core at their center [37]. The small sizes of IQ-1S-treated spheroids could prevent central necrosis and result in a decrease in PI intensity due to its dependence on the spheroid volume captured in the focal plane upon imaging [38]. Therefore, using this technique to measure the cytotoxic effect of anticancer drugs in 3D objects may yield underestimated results.

Tetramethylrhodamine methyl ester perchlorate (TMRM), a fluorescent dye that accumulates in mitochondria in response to the negative membrane potential has been previously used to comparatively assess mitochondrial membrane potential in 3D multicellular structures [39,40]. Loss of mitochondrial membrane potential is an early event in apoptosis, making TMRM a useful tool for the early identification of cells at this stage of the process [41]. We found that the TMRM fluorescence parameter did not change in 7-day-old 3D objects (Table 2), confirming the absence of the effect of the JNK inhibitor on the mitochondrial apoptotic pathway.

**Figure 2:**
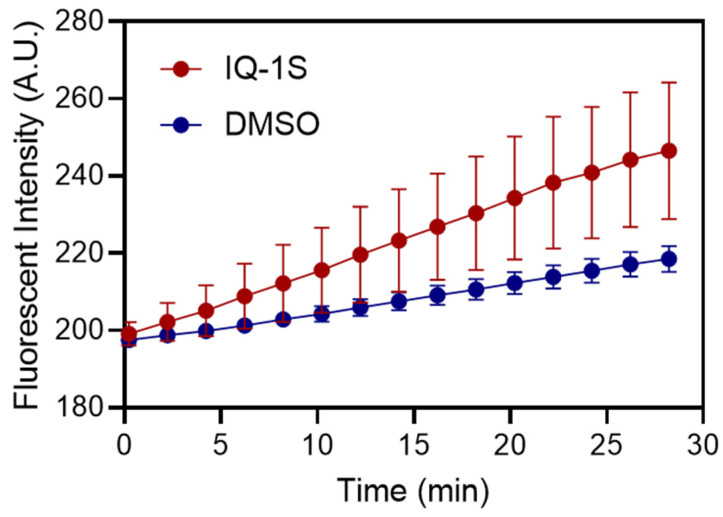
Kinetics of intracellular FDA hydrolysis in MCF7 spheroids. Multicellular 7-day-old MCF7 spheroids in a hydrogel matrix were exposed to FDA solution and imaged at 2 min-intervals. The FI versus time for 3D microtissues grown in the presence IQ-1S (20 µM, red circles) or without compound (0.1% DMSO, blue circles). The values represent mean ± S.D. at each time point.

The FDA staining was used to assess cell viability because the rate of accumulation of fluorescent hydrolysis products of this esterase substrate in cells reflects both the enzymatic activity required for hydrolysis and the integrity of the cell membrane required to retain the product. No hydrolyzed fluorescein accumulated, and no intracellular fluorescence was detected in membrane-compromised cells, such as dead, PI-positive cells. As a result, the rate of FDA hydrolysis was significantly higher (*p* < 0.001) in spheroids treated with IQ-1S than in control samples treated with DMSO (Figure 2). This may indicate an activated state of cells or accelerated cellular metabolism in spheroids treated with the JNK inhibitor. Indeed, the conversion of the substrate (FDA) from extracellular to intracellular levels depends primarily on active and passive mechanisms of cell membrane permeability [42]. The state of the cell membrane and cytoplasm changes structurally and functionally under the influence of pharmacological agents. This can lead to a change in the effective concentration of the intracellular substrate required for maximum enzyme–substrate interaction [43].

**Figure 3:**
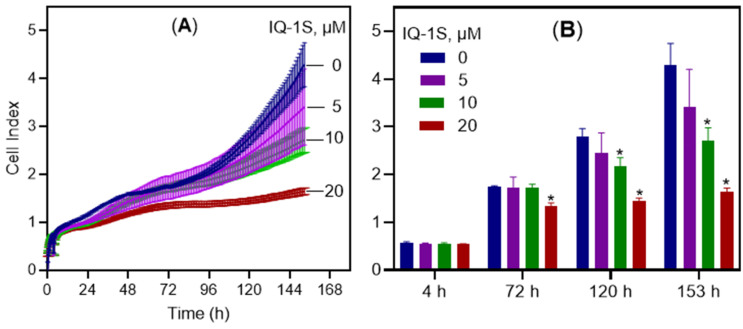
Time-dependent changes in cell index values and the average cell index values for MCF7 cells after the treatment with IQ-1S as compared to the negative control (1% DMSO). Panel (**A**) displays cell index values recorded at 15 min intervals, while panel (**B**) presents these values at selected time points. The values represent mean ± S.D., *n* = 4. * *p* < 0.05 in comparison with the control group (0 µM IQ-1S or 1% DMSO).

We also assessed the effect of IQ-1S on MCF7 cells grown for 153 h (6.4 days) as a monolayer (2D culture) using the iCELLigence system. The xCELLigence is a technology that gives the possibility to measure the cellular growth in real-time by measuring the net adhesion of cells on a custom-designed gold electrode following the changes in electrical impedance [44]. Figure 3 shows the normalized cell index of MCF7 cells treated with varying concentrations of IQ-1S (5, 10, and 20 µM) relative to the negative control (1% DMSO). Treatment was initiated 4 h after cell seeding, and the cell index was normalized at the time of compound addition. A delay in cell growth became apparent as early as 20 h post-treatment, with maximal suppression of proliferation observed at 153 h (Figure 3B).

**Figure 4:**
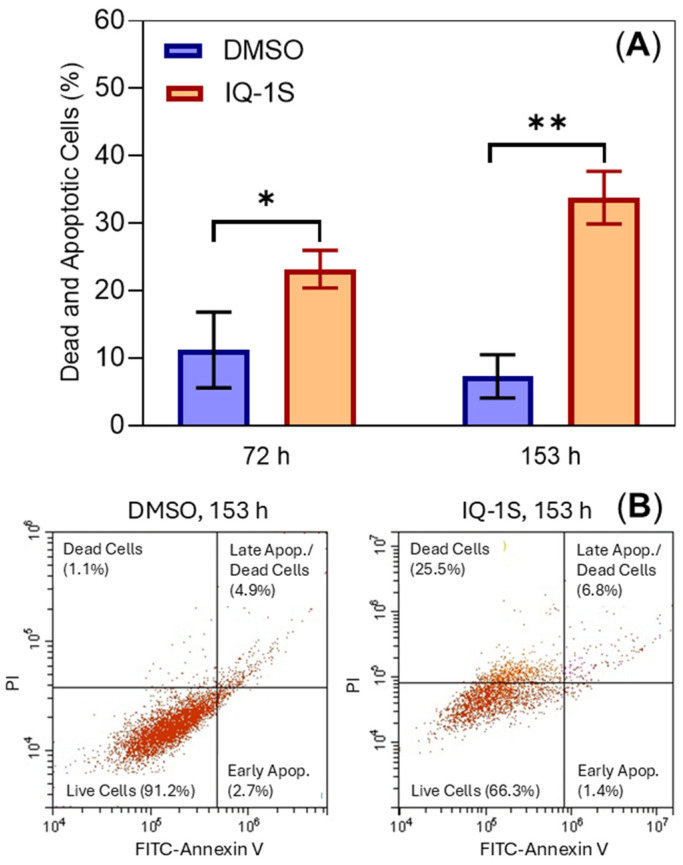
IQ-1S induces cell death of MCF cells grown as a monolayer (2D culture) assessed by flow cytometry. Panel (**A**) displays a percentage of dead and apoptotic cells after 72 and 153 h of treatment with IQ-1S (20 µM) as compared to the negative control (1% DMSO). The values represent mean ± S.D., *n* = 3. * *p* < 0.05 and ** *p* < 0.01 in comparison with the control group. MCF7 cell apoptosis measured by annexin V/PI staining is shown in panel (**B**) of representative flow cytometry data for control (1% DMSO) and IQ-1S (20 μM)-treated cells at 153 h post-compound application (panel (**B**)). The bivariant plots on panel B display individual cells or events, with each event plotted according to the PI and FITC-annexin fluorescence intensity parameters. Each plot shows quadrants with dead and apoptotic cells: viable cells (lower left quadrant) were negative for both annexin V and PI, apoptotic cells were positive for annexin V staining and negative for PI, and late apoptotic/dead cells were positive for both annexin V and PI (upper right quadrant).

When MCF7 cells grown in 2D culture were treated with IQ-1S (20 µM) for 72 and 153 h, a significant increase in cell death was observed by flow cytometry using fluorescein isothiocyanate (FITC)-annexin V/PI staining. Different stages of apoptosis (early and late apoptosis, and necrosis) were identified, and a significant increase in all stages was observed (Figure 4). In addition, the data were supported by a fluorescence microscopy with visual counting of green (apoptotic cells) and red (necrotic cells) fluorescence. The percentage of dead cells was significantly (*p* < 0.01) increased in MCF7 cells treated with IQ-1S (20 μM) for 153 h: 16.7 ± 0.8% vs. 1.1 ± 0.5% in DMSO-treated cells.

Qing Li et al. showed that the JNK inhibitor SP600125 significantly inhibited the growth of MCF7 breast cancer cells in monolayer 2D culture [17]. Our studies using compound IQ-1S in both 2D and 3D cultures support the idea that JNK inhibitors may be potential drugs for the treatment of breast cancer. It should be noted that TMRM staining of IQ-1S-treated spheroids revealed no difference with control objects (Table 2), confirming the absence of an effect of the JNK inhibitor on the mitochondrial-dependent (intrinsic) early apoptotic pathway. However, IQ-1S-treated MCF7 cells in 2D culture showed an apoptosis as assessed by flow cytometry (Figure 4) and fluorescence microscopy. Indeed, the resistance/sensitivity of tumor cells to the antitumor drugs may differ in 3D culture compared to monolayer [45]. The reason for the different cell sensitivities to this kinase inhibitor is unknown. One possible explanation could be cell polarization within the spheroids and the different sensitivities of these polarized cells to this compound. Specifically, Cui Li et al. reported that 3D culturing of MCF7 and MCF10A cells resulted in the formation of an acinus, an equivalent of apical–basolateral polarity in vitro [46], and the JNK inhibitor SP600125 stopped acinus formation and cell polarization in the 3D culture of MCF10A cells [47].

**Figure 5:**
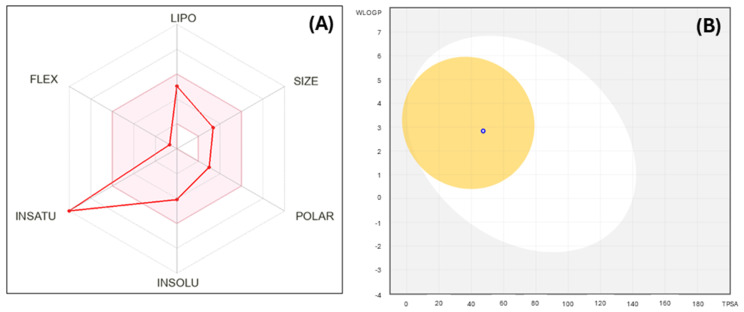
Panel (**A**): bioavailability radar plot for IQ-1S. The plot depicts LIPO (lipophilicity), SIZE (molecular weight), POLAR (polarity), INSOLU (insolubility), INSATU (insaturation), and FLEX (rotatable bond flexibility) parameters. Panel (**B**): the SwissADME boiled-egg diagram for compound IQ-1S. IQ-1S was predicted to be a substrate of P-glycoprotein (PGP+, blue open dot) and to have potential gastrointestinal absorption (HIA, dot inside the white oval) and blood–brain barrier (BBB) permeability (dot inside the yellow circle).

We also constructed a bioavailability radar plot that depicts the drug-likeness of compound IQ-1S. Six physicochemical properties were considered: lipophilicity (LIPO), size, polarity (POLAR), insolubility (INSOLU), flexibility (FLEX), and unsaturation (INSATU). The physicochemical range on each axis is represented by the pink area, which the molecule’s radar diagram should completely fall within to achieve high predicted bioavailability [48]. We found that IQ-1S has generally satisfactory ADME properties and high bioavailability (Figure 5A, Supplementary Table S1), including a topological polar surface area (TPSA) of 47.37 Å^2^ suggesting good permeability, and a bioavailability score of 0.55. The value of “INSATU” is outside the region due to the increased unsaturation of the molecule, which is, however, typical for some drug candidates, such as the well-known JNK inhibitor SP600125 [36,49]. The boiled-egg diagram (Figure 5B) demonstrated the potential gastrointestinal absorption and blood–brain barrier (BBB) permeability of the test compound. In addition, it was predicted that IQ-1S is a P-glycoprotein substrate, so it can be actively pumped out of cells [50]. It is noteworthy that the compound does not violate important bioavailability criteria such as Lipinski’s rule of five, Gauche and Weber filters (Supplemental Table S1). These findings suggest favorable pharmacokinetic properties despite some potential for efflux-mediated limitations. Our predictions are supported by experimental data in rats, where the corresponding oxime IQ-1 demonstrated a high apparent volume of distribution (879 L/kg after 50 mg/kg oral dose of the compound) indicating extensive tissue penetration, and dose proportionality only at higher doses (50–100 mg/kg), consistent with the saturation of transport processes [51,52].

Although the concentration of IQ-1S for inhibiting spheroid growth in vitro is relatively high (20 µM), previous studies have demonstrated its efficacy in various in vivo models at achievable and well-tolerated doses, highlighting its therapeutic potential. In partial, the serum concentration of IQ-1S was around 10–12 μM during the first 10 min after intraperitoneal (*i.p.*) administration at a dose of 30 mg/kg [35]. Moreover, the administration of IQ-1S (*i.p*., daily at a dose of 50 mg/kg) for 1 month was well-tolerated and did not appear as a toxic effect in mice [53]. The therapeutic effects of IQ-1S were found when the compound IQ-1S or its derivatives IQ-1 and IQ-1L) were administered *i.p.* at dose 12–50 mg/kg in rodent models of cerebral ischemia and collagen-induced arthritis, resulting in significant neuroprotection and reduced joint inflammation [53,54,55], suggesting effective tissue accumulation despite its recognition as a P-glycoprotein substrate, which may limit intracellular concentrations in some tissues by promoting efflux [56]. While P-glycoprotein-mediated efflux could potentially reduce IQ-1S accumulation in certain multidrug-resistant cancer cells or at the blood–brain barrier, the compound’s overall ADME profile included moderate solubility and no violations of Lipinski’s rules. Furthermore, localized delivery systems such as electrosprayed poly(lactic-co-glycolic acid) particles and poly(ε-caprolactone) scaffolds described in related studies have shown that they can provide sustained release and targeted accumulation of IQ-1S and its derivatives [57,58]. A direct effect of IQ-1S and its lithium salt IQ-1L on JNK activity was also demonstrated using phosphorylation assay in leukemic cell line MonoMac-6 [35,54] as well as inhibitory effect of IQ-1S on c-Jun phosphorylation in vivo [59]. These findings, combined with the compound’s favorable ADME properties, suggest that IQ-1S holds promise for clinical applications, and ongoing chemical modifications of related analogs could further optimize its potency if necessary [32].

Previously, JNK inhibitors SP600125 and JNK-IN-8 significantly suppressed the growth of MCF7 and MCF10CA1a breast cancer cells [16,17,60]. Because different models together with our study were previously used to test this activity, it is difficult to draw a definitive conclusion about which compound is more suitable for use as an antitumor agent. Further studies are needed to determine their antitumor efficacy in comparable in vitro and in vivo tumor growth models, as well as direct evidence of JNK signaling pathway inhibition (e.g., c-Jun phosphorylation analysis) in tumor spheroids.

## 3. Materials and Methods

### 3.1. Materials, Reagents, and Studied Compound

The low melting agarose was obtained from Cambrex Bio Science Rockland, Inc. (Rockland, ME, USA). A Sylgard 184 Kit was purchased from Dow Corning Corp. (Midland, MI, USA). PI, TMRM, and DMSO were purchased from Sigma-Aldrich (St. Louis, MO, USA). Dulbecco’s modified Eagle medium (DMEM), heat-inactivated fetal calf serum, penicillin, streptomycin, glutamine, sodium pyruvate, and phosphate-buffered saline were obtained from Biological Industries (Kibbutz Beit Haemek, Israel). Six well glass-bottom plates were purchased from In Vitro Scientific (Sunnyvale, CA, USA).

Sodium salt of 11*H*-indeno[1,2-*b*]quinoxalin-11-one oxime (IQ-1S) was synthesized as described previously [34]. The chemical structure was confirmed by the methods of mass spectrometry and nuclear magnetic resonance (NMR). The purity of the sample was 99.9%. According to our observations and NMR data, the compound studied is stable and its tetracyclic oxime scaffold remains unchanged in an aqueous DMSO solution (50% *v*/*v*) for one month.

### 3.2. Hydrogel Microchamber Array (hMCA)

hMCA was designed and fabricated as described previously [39,40]. Briefly, an array of square-bottom pyramid-shaped microchambers was obtained from GeSiM mbH (Großerkmannsdorf, Radeberg, Germany) and used for the production of the polydimethylsiloxane (PDMS) stamp with a negative microchamber array. Fabrication of the hMCA was performed in the specially modified commercial six-well glass bottom plates. Warm agarose was dripped on the surface of the plate’s glass bottom, and a pre-heated PDMS stamp gently placed over it. The system was incubated at room temperature, followed by incubation at 4 °C for agarose gelation. At the culmination of the gelation process, the PDMS stamp was peeled off, leaving agarose gel patterned with a square geometry of microchambers (from 400 to 800 in each macro well) with 90 μm on each side and about 100 μm in depth. The imaging plate, consisting of an optical bottom patterned with hMCA was UV sterilized and stored at 4 °C in humidified atmosphere until use.

### 3.3. Cell Culture and Microtissue Formation

Human breast cancer MCF7 cell line was obtained from American Type Culture Collection (Manassas, VA, USA). MCF7 cells were maintained in DMEM medium, supplemented with 10% heat-inactivated fetal calf serum, 100 U/mL penicillin, 100 µg/mL streptomycin, 2% glutamine, and 2% sodium pyruvate (complete medium). Cells were maintained in a completely humidified atmosphere with 5% CO_2_ at 37 °C. Before use, the exponentially growing cells were collected by trypsinization, washed and re-suspended at appropriate concentrations in fresh complete medium. Cell suspension (50 µL, 1–3 × 10^5^ cells/mL) was loaded onto hMCA and then set aside to allow cellular/multicellular structure formation in each individual microchamber. Three-dimensional microtissues were grown in the present or without IQ-1S or vehicle (0.1% DMSO) added once/three times during the first three days of growth and monitored until 7 days.

### 3.4. Cellular Object Staining

All live fluorescent staining procedures were performed in situ when multicellular microstructures are within the hMCA and were measured at the appropriate wavelength. Cell viability within each microtissue was determined by staining with PI at a final concentration of 3.7 μM. Microtissue viability was calculated as the percentage of fluorescent area within the entire CSA of each individual 3D object.

The integrity of the cytoplasmic membrane and intracellular enzymatic activity were determined by FDA staining (1.2 μM) as a kinetic test in single-cell microtissues. The rate of FDA hydrolysis by intracellular non-specific esterase was measured and calculated based on repeated periodic measurements (10 times with an interval of 2 min) and presented as a linear dependence.

Mitochondrial trans-membrane potential was measured by TMRM staining (final concentration 12.5 nM) in complete medium at 37 °C under a humidified atmosphere with 5% CO_2_ for 1 h. The results are presented as fluorescent intensity’s (FI) CV (coefficient of variation, i.e., standardized S.D., %) in order to avoid the FI’s variability created both by TMRM staining per individual experiment and by the significant difference in the sectional area between single 3D objects.

### 3.5. Imaging System and Operating Software

Images were acquired using a motorized Olympus inverted IX81 microscope (Tokyo, Japan). The microscope is equipped with a sub-micron Marzhauser-Wetzlar motorized stage type SCAN-IM, with an Lstep controller (Wetzlar-Steindorf, Wetzlar, Germany) and a filter wheel including fluorescence cubes suitable for specific dyes. All filters were obtained from Chroma Technology Corporation (Brattleboro, VT, USA). A 14-bit cooled, highly sensitive ORCA II C4742-98 camera (Hamamatsu, Japan) was used for imaging. Most of the images were taken with a ×10 magnification objective. The complete microscope system was enclosed in an incubator which provided a temperature of 37 °C and humidified atmosphere containing 5% CO_2_ (Life Imaging Services, Basel, Switzerland), allowing monitoring over long periods. Image acquisition was performed continuously at each experimental step.

### 3.6. Image Analysis

Olympus CellR software was used for image analysis (Tokyo, Japan). For optical data acquisition and analysis, each set of image acquisitions was initiated by first acquiring the bright field image of a chosen view field, followed by the acquisition of several fluorescent images, one for each fluorescent probe, taken at a different preset time points. A series of regions on the hMCA were chosen and saved in a list of positions on the motorized stage. The initial cell distribution in each microchamber in those regions was imaged. For continuous monitoring of the microtissue formation process, the imaging system was programmed to take images of each saved position automatically, with consistent time intervals between each image, and the 6-well plate was either left on the microscope stage or placed in an external incubator and then returned to the microscope, verifying that the same regions were scanned, and images of the same spheroids taken.

Individual spheroids were defined as regions of interest (ROIs), and their CSA was outlined on the bright field image. Morphometric parameters of spheroids were extracted by image-processing algorithms of bright-field microscopy. Then, on each fluorescent wide-field image, ROIs were determined by mapping those outlines on the interrogated fluorescent field image. Next, the fluorescent background, determined by averaging the fluorescence intensity detected by camera pixels found between the outlined regions, was subtracted from the fluorescent image. It should be emphasized that background signal determination and subtraction were performed separately for each of the acquired fluorescent field images. The fluorescence images were then thresholded, and for each region of interest (all pixels within the CSA of the object that were within the threshold boundaries) the average fluorescence intensity was obtained and the percentage of the area of these pixels (all fluorescent signals in the ROI) relative to the total CSA of the region of interest was calculated.

### 3.7. Real-Time Cell Analysis Using the iCELLigence System

In the real-time cell analysis (RTCA) system, cytotoxicity is measured by cellular impedance readout as cell index (CI) to monitor real-time changes in cell number. The iCELLigence system (Agilent Technologies, Santa-Clara, CA, USA) was used according to the manufacturer’s instructions. The CI was obtained by measuring the change in the electrical impedance in the presence and absence of cells in the wells. The MCF7 cell line was inoculated keeping the cell number as 1 × 10^4^ cells/well, on 8-well plates of the iCELLigence system. Afterward, the proliferation, attachment and spreading of the cells were monitored in each well of e-plates every 60 min and analyzed with RTCA Software 1.2. Four hours after cell transplantation, cells in the logarithmic growth phase were treated with various concentrations of IQ-1S in DMSO (final DMSO concentration: 1%) and monitored in real time—every 15 min for the first 12 h, then hourly—up to 168 h. Cells grown in growth medium containing 1% DMSO were used as the control. All experimental steps were conducted at 37 °C in a 5% CO_2_ atmosphere.

### 3.8. Apoptosis Detection with Annexin V-FITC/Propidium Iodide

Apoptosis was measured using Annexin V-FITC/PI Apoptosis kit (Cat. No: E-CK-A211; Elabscience, Wuhan, China) on days 3 and 7 of IQ-1S treatment or 1% DMSO as a positive control. Briefly, 1 × 10^6^ cells were trypsinized with trypsin-ethylenediaminetetraacetic acid (EDTA), washed with phosphate-buffered saline and collected in 500 μL annexin V binding buffer, stained with 5 μL annexin V and 5 μL PI according to the manufacturer’s protocol. After 15 min of incubation at room temperature, samples were analyzed on a CytoFlex flow cytometer (Beckman Coulter, Brea, CA, USA). Viable cells (lower left quadrant) were stained negatively with both annexin V and PI, apoptotic cells were stained positively with both annexin V and PI, and dead/late apoptotic cells were stained positively with both annexin V and PI (upper right quadrant) (see Figure 4B).

To assess cell death using fluorescence microscopy, cells were stained with annexin V-FITC and PI, as described above. Cells were counted in 10 fields of view using an EVOS M7000 microscope imaging system (Thermo Fisher Scientific, Waltham, MA, USA). Cells bound to annexin V-FITC emitted green fluorescence and were considered apoptotic, while cells stained red (PI) were considered necrotic.

### 3.9. Statistical Analysis

Each test was performed at least twice (2 macro-wells). A total of 10–12 images were acquired from different areas of the hMCA, resulting in approximately 120 individual spheroids per macro-well and approximately twice that number for a test. The mean and standard deviations of each measured parameter were calculated for the different spheroid populations studied. Comparisons between groups were performed using Student’s *t*-test, with differences considered statistically significant at *p* < 0.05 for Gaussian-distributed groups and analysis of variance (ANOVA) for more than two groups.

### 3.10. Molecular Modeling

The physicochemical properties of IQ-1S were computed using SwissADME (http://www.swissadme.ch, accessed on 1 August 2025) [48].

## 4. Conclusions

In conclusion, we report the morphological and functional characterization of breast cancer spheroids treated with the JNK inhibitor IQ-1S. The CSA of 7-day-old 3D objects was significantly smaller when treated with IQ-1S (20 μM), compared to DMSO-treated control samples. However, the CSA of 7-day-old 3D objects was not significantly different from control samples when grown under 2 μM IQ-1S conditions. Cell death assessed as the percentage of PI-positive area in the entire 3D object area, and mitochondrial transmembrane potential assessed as CV (%), were similar in control untreated and IQ-1S-treated samples. The rate of FDA hydrolysis was significantly higher in 3D spheroids grown under 20 μM IQ-1S treatment compared to controls. MCF7 cells in 2D monolayer culture treated with IQ-1S showed significant inhibition of proliferation and increased apoptosis. In conclusion, our in vitro studies and in silico evaluation showed that IQ-1S has satisfactory ADME properties and high potential for use in preclinical tumor growth models.

## Figures and Tables

**Table 1:** Growth of 3D spheroid MCF7 microtissues.

Characteristics of 3D Objects	IQ-1S	Vehicle	*p*-Value ^1^
Cell seeded (cells/3D object)	9.0 ± 3.4	9.7 ± 3.5	0.66
CSA of 3D objects at 24 h (mm^2^)	0.0013 ± 0.0005	0.0015 ± 0.0006	0.44
CSA of 3D objects at 168 h (mm^2^)	0.0036 ± 0.0014	0.0076 ± 0.0037	0.003
*p*-value (CSA at 24 vs. 168 h)	0.0001	0.0001	

^1^ *p*-value for the CSA of spheroids treated with 20 µM IQ-1S compared to untreated (0.1% DMSO solution). Results are presented as mean ± standard deviation (S.D.). *p*-value was calculated using analysis of variance (ANOVA).

**Table 2:** Vital parameters of 7-day-old MCF7 spheroids treated with IQ-1S (20 µM) or vehicle (0.1% DMSO).

Fluorescent Dye and Parameter	IQ-1S	Vehicle	*p*-Value
PI (% of entire CSA)	7.2 ± 4.4	2.5 ± 1.3	0.13
TMRM FI as CV (%)	123.4 ± 29.6	124.2 ± 33.7	0.9

TMRM FI, fluorescence intensity of tetramethylrhodamine methyl ester. Results are presented as average mean ± S.D. *p*-value was calculated using ANOVA.

## Data Availability

Data are contained within the article.

## Supplementary Materials

The following supporting information can be downloaded at https://www.mdpi.com/article/10.3390/molecules30214278/s1, Table S1: Structural characteristics and predicted ADME properties of JNK inhibitor IQ-1S.
