# Supplementary material for: c-Jun N-Terminal Kinase (JNK) Inhibitor IQ-1S as a Suppressor of Tumor Spheroid Growth
Source: Molecules. 2025 Nov 3;30(21):4278. doi: 10.3390/molecules30214278 (PMC12609690; doi:10.3390/molecules30214278)
Supplement: Supplementary file 1 [file molecules-30-04278-s001.zip › molecules-3963945-supplementary.pdf]

Supplementary Materials

for

c-Jun N-Terminal Kinase (JNK) Inhibitor IQ-1S as a Suppressor of Tumor Spheroid Growth

Elena Afrimzon [1,2], Mordechai Deutsch [1], Maria Sobolev [1], Naomi Zurgil [1], Andrei I. Khlebnikov [3,*], Mikhail A. Buldakov [4] and Igor A. Schepetkin [5,*]

[1] The Biophysical Interdisciplinary Jerome Schottenstein Center for the Research and Technology of the Cellome, Physics Department, Bar Ilan University, Ramat Gan 5290002, Israel; afrimzone@gmail.com (E.A.); motti.jsc@gmail.com (M.D.); eweryday@yahoo.com (M.S.): zurgiln@gmail.com (N.Z.)

[2] Institute of Nanotechnology and Advanced Materials, The Mina and Everard Goodman Faculty of Life Sciences, Bar-Ilan University, Ramat Gan 5290002, Israel

[3] Kizhner Research Center, Tomsk Polytechnic University, Tomsk 634050, Russia

[4] Cancer Research Institute, Tomsk National Research Medical Center, Russian Academy of Sciences, Tomsk 634050, Russia; buldakov@oncology.tomsk.ru

[5] Department of Microbiology and Cell Biology, Bozeman, MT 59717, USA

* Correspondence: aikhl@chem.org.ru (A.I.K.); igor@montana.edu (I.A.S.)

Supplementary Table S1. Structural characteristics and predicted ADME properties of JNK inhibitor IQ-1S.

Parameter	Value of ADME Parameter
Canonical SMILES	[Na]O/N=C\1/c2ccccc2c2c1nc1ccccc1n2
Formula	$C_{15}H_{8}N_{3}NaO$
Molecular Weight	269.23
#Heavy Atoms	20
#Aromatic heavy atoms	16
Fraction Csp3	0
#Rotatable Bonds	1
#H-bond Acceptors	4
#H-bond Donors	0
MR	71.7
TPSA	47.37
XLOGP3	3.28
WLOGP	2.84
MLOGP	1.62
Silicos-IT Log P	2.1
Consensus Log P	1.97
ESOL Log S	-4.1
ESOL Solubility (mg/ml)	2.13E-02
ESOL Solubility (mol/l)	7.91E-05
ESOL Class	Moderately soluble
Ali Log S	-3.95
Ali Solubility (mg/ml)	3.02E-02
Ali Solubility (mol/l)	1.12E-04
Ali Class	Soluble
Silicos-IT LogSw	-5.97
Silicos-IT Solubility (mg/ml)	2.90E-04
Silicos-IT Solubility (mol/l)	1.08E-06
Silicos-IT Class	Moderately soluble
GI Absorption	High
BBB Permeant	Yes
P-gp Substrate	Yes
CYP1A2 Inhibitor	Yes
CYP2C19 Inhibitor	No
CYP2C9 Inhibitor	No
CYP2D6 Inhibitor	Yes

Supplementary Table S1 (continued)

CYP3A4 Inhibitor	Yes
log Kp (cm/s)	-5.61
Lipinski #Violations	0
Ghose #Violations	0
Veber #Violations	0
Egan #Violations	0
Muegge #Violations	0
Bioavailability Score	0.55
PAINS #Alerts	0
Brenk #Alerts	2
Leadlikeness #Violations	0
Synthetic Accessibility	2.87
